# Utility of the History and Physical Examination in the Detection of Acute Coronary Syndromes in Emergency Department Patients

**DOI:** 10.5811/westjem.2017.3.32666

**Published:** 2017-05-03

**Authors:** Zachary DW Dezman, Amal Mattu, Richard Body

**Affiliations:** * University of Maryland School of Medicine, Department of Emergency Medicine, Baltimore, Maryland; † Manchester Royal Infirmary, Department of Emergency Medicine, Manchester, United Kingdom

## Abstract

Chest pain accounts for approximately 6% of all emergency department (ED) visits and is the most common reason for emergency hospital admission. One of the most serious diagnoses emergency physicians must consider is acute coronary syndrome (ACS). This is both common and serious, as ischemic heart disease remains the single biggest cause of death in the western world. The history and physical examination are cornerstones of our diagnostic approach in this patient group. Their importance is emphasized in guidelines, but there is little evidence to support their supposed association. The purpose of this article was to summarize the findings of recent investigations regarding the ability of various components of the history and physical examination to identify which patients presenting to the ED with chest pain require further investigation for possible ACS.

Previous studies have consistently identified a number of factors that increase the probability of ACS. These include radiation of the pain, aggravation of the pain by exertion, vomiting, and diaphoresis. Traditional cardiac risk factors identified by the Framingham Heart Study are of limited diagnostic utility in the ED. Clinician gestalt has very low predictive ability, even in patients with a non-diagnostic electrocardiogram (ECG), and gestalt does not seem to be enhanced appreciably by clinical experience. The history and physical alone are unable to reduce a patient’s risk of ACS to a generally acceptable level (<1%).

Ultimately, our review of the evidence clearly demonstrates that “atypical” symptoms cannot rule out ACS, while “typical” symptoms cannot rule it in. Therefore, if a patient has symptoms that are compatible with ACS and an alternative cause cannot be identified, clinicians must strongly consider the need for further investigation with ECG and troponin measurement.

## CASE REPORT

A 50-year-old man presented to the emergency department (ED) because he was experiencing chest pain after eating a large meal. He had discomfort in his central chest, which he described as “like indigestion.” The pain did not radiate, was not associated with other symptoms, and resolved spontaneously after 30 minutes. The patient had no relevant medical history and no risk factors for ischemic heart disease. An electrocardiogram (ECG) obtained soon after his arrival in the ED was normal.

Is acute coronary syndrome (ACS) likely to be the cause of this man’s chest pain? Can we use any features of his history and physical examination to determine the likelihood of ACS?

## INTRODUCTION

Ischemic heart disease remains the leading cause of death in the United States, accounting for a quarter of all deaths.^1^,^2^ Accurate recognition of acute myocardial infarction (AMI) in the ED is crucial, as the mortality rate of patients with missed AMI is at least double that of similar patients who are accurately diagnosed.^3^ Missed AMI is also one of the most common reasons for medical malpractice claims in the U.S. 4–6

Chest pain is the second most common reason for ED visits, accounting for 5.4% of all presentations, and many of these patients are admitted for evaluation for ACS.^7^ The costs involved are staggering: In the U.S. in 2011, the cost for admitting patients with chest pain totaled $11.5 billion, representing 3% of the nation’s healthcare expenditures.^8^ Although the mortality rate for admitted patients is lower than for those whose AMI goes undetected, hospital admission presents its own risks, including infection and procedural complications.^9^ A recent retrospective analysis revealed that there was a very low incidence of short-term adverse cardiac events in chest pain patients who were hospitalized after an ED workup determined they were low risk (i.e,, patients with non-concerning vital signs, non-ischemic ECG, and two negative troponins taken in the ED between 60 and 420 minutes apart).^10^ This finding suggests that not every patient with chest pain will benefit from a full admission and that risk stratification can be improved. In this article, we review the evidence regarding the utility of the patient’s history and physical examination in determining the risk of ACS in patients who present to EDs with chest pain.

## UTILITY OF THE HISTORY OF PRESENT ILLNESS AND PHYSICAL EXAMINATION

Electrocardiography and troponin testing are considered the cornerstones of the diagnosis of AMI and ACS, but they are both insensitive at the time of ED evaluation.^11^,^12^ The history of presenting illness (HPI) and physical examination provide an immediate source of information by which emergency physicians can stratify patients according to the need for further workup. Multiple guidelines have supported this approach,^13^,^14^ but only a few groups have examined which features of the HPI are most correlated with cardiac causes in undifferentiated ED patients with chest pain. Of chief importance was an exploration of typical (i.e., severe and acute-onset chest pain, most often left-sided, provoked by effort and accompanied by anxiety, shortness of breath, and a choking sensation)^15^ and atypical symptoms. Goodacre et al. prospectively enrolled 893 patients presenting with chest pain between 1999 and 2000.^16^ They defined AMI according to guidelines from the World Health Organization (WHO) and ACS as one of the following: a positive stress test; a positive troponin level; an ischemic pattern on ECG; or subsequent AMI, death by cardiac cause, or revascularization within six months. Multivariate analysis found that AMI was most closely associated with chest pain that radiated to the shoulder (odds ratio [OR]=5.7, 95% confidence interval [CI] [1.5–21.4], p=0.009), radiated to both arms (OR=4.9, 95% CI [1.3–19.4], p=0.02), or was exacerbated with exertion (OR=3.3, 95% CI [1.3–8.4], p=0.014). ACS was most closely associated with radiation to the shoulder (OR=5.2, 95% CI [2.0–13.4], p=0.0008), left arm (OR=2.1, 95% CI [1.0–4.4], p=0.042), or both arms (OR=4.8, 95% CI [1.8–13.2], p=0.002) or pain that was exertional (OR=2.4, 95% CI [1.3–4.5], p=0.005). The same analysis found that the presence of chest wall tenderness decreased the likelihood of AMI (OR=0.2, 95% CI [0.05–0.97], p=0.045).

Milner and associates prospectively recorded the symptomatology and medical history of 531 patients presenting to an ED with chest pain. Their primary focus was on age-related differences in the presentation of ACS. Of the patients diagnosed with ACS, those older than 70 had a higher burden of comorbidities than did those younger than 70. The comorbidities included a history of MI (51% vs 31%, p=0.038), coronary heart disease (50% vs 73%, p=0.001), hypercholesterolemia (45% vs 32%, p=0.045), and heart failure (28% vs 10%, p=0.001). Further analysis showed that chest pain (OR=2.47, 95% CI [1.37–4.42], p=0.002), radiation to the arm (OR=1.78, 95% CI [1.03–3.09], p=0.040), and fatigue (OR=2.52, 95% CI [1.10–5.81], p=0.29) were all positively associated with ACS in younger subjects, yet none of these factors was significant in older subjects. An increasing number of typical symptoms was associated with ACS in those under 70 years of age. However, the “typicality” of presentation had no association with ACS in older patients. These findings suggest that older patients with ACS have a higher burden of traditional cardiac risk factors but present with few of the traditional symptoms.

In 2004, two internists conducted a systematic review of the literature to assess bedside findings useful in diagnosing AMI in patients with chest pain.^18^ Their review included studies of patients admitted to inpatient wards and intensive care units as well as undifferentiated ED patients. This review included patients with stable cardiac disease in addition to a group that had experienced MI, but it did not examine the entire spectrum of ACS. Among patients with acute chest pain, the features that best predicted AMI were right arm or shoulder pain (LR+=4.7, 95% CI: 1.9–12), an S3 gallop (LR+=3.2, 95% CI [1.6–6.5]), and either a history (LR+=2.1, 95% CI [1.8–2.5]) or a finding of diaphoresis (LR+=2.9, 95% CI [1.3–6.6]). Adding an ECG was helpful, as new ST-elevations (LR+=22, 95% CI [16–30]), new ST-depressions (LR+=4.5, 95% CI [3.6–5.6]), and new Q waves (LR+=22, 95% CI [7.6–62]) all strongly predicted an AMI. The factors that decreased the likelihood of AMI were a normal ECG (LR+=0.2; 95% CI [0.1–0.3]) and reproducible chest wall tenderness (LR+=0.3; 95% CI [0.2–0.4]). Chest pain that was pleuritic (LR+=0.2; 95% CI [0.2–0.3]), sharp (LR = 0.3; 95% CI [0.2–0.5]), or positional (LR = 0.3; 95% CI [0.2–0.5]) also lowered the likelihood of AMI.

Bruyninckx and associates conducted a meta-analysis of patients with chest pain, looking for features that predicted AMI or ACS.^19^ The studies included undifferentiated ED patients, admitted patients, and those being observed in chest pain units. They found very few features with LR≥3 or LR≤0.4. Pain in the right arm or shoulder was suggestive of ACS in both selected (LR+=3.78, 95% CI [2.17–6.60]) and undifferentiated patients (LR+=3.80, 95% CI [1.12–12.91]). The next most-predictive features were severe pain (LR+=1.68, 95% CI [1.40–2.02]) and neck pain (LR+=1.44, 95% CI [1.12–1.86]) in undifferentiated patients. The feature most closely associated with an alternative diagnosis was chest wall tenderness (LR+=0.17, 95% CI [0.11–0.28]).

Body and colleagues examined 804 patients who sought treatment in a university-affiliated ED in the United Kingdom for complaints suggestive of cardiac chest pain^20^ (Table). Their primary outcome was AMI,^21^ with a secondary outcome of an adverse cardiac event within the next six months. An adverse cardiac event was defined as death (from all causes), AMI, angiographic evidence of new stenosis ≥50% not amenable to intervention, or the need for revascularization within six months after the index ED visit. Revascularization was defined as a non-elective percutaneous coronary intervention or bypass grafting. AMI was diagnosed in 18.6% during the index ED visit, and 22.9% had experienced an adverse cardiac event by the time of follow-up six months later. The features of the HPI and physical exam that were most associated with AMI were observed diaphoresis in the ED (LR+=6.39, 95% CI [4.38–9.33]), reported vomiting (LR+=3.09, 95% CI [1.89–5.05]), and pain radiation to both arms/shoulders (LR+=2.58, 95% CI [1.53–4.34]) or to the right arm/shoulder (LR+=2.31, 95% CI [1.52–3.53]). Hypotension was also associated with AMI (LR+=2.92, 95% CI [1.34–6.37]), but it was rare (occurring in 6.8% of subjects with AMI). For all subjects with AMI or adverse cardiac events at six-month follow-up, the most predictive signs and symptoms were observed diaphoresis (LR+=5.11, 95% CI [3.42–7.63]), reported vomiting (LR+=2.97, 95% CI [1.82–4.85]), and radiation of pain to both shoulders/arms (LR+=2.57, 95% CI [1.55–4.29]) or to the right arm or shoulder (LR+=2.22, 95% CI [1.47–3.34]). Hypotension continued to be an insensitive (7.6%) but strong predictor of a cardiac cause of chest pain (LR+=4.93, 95% CI [2.21–10.98]). Many of the individual risk factors and features of the HPI had areas under their receiver operating curves very close to 0.5, making them only slightly better than a coin flip when determining whose chest pain had a cardiac cause. The authors concluded that many typical features of AMI and ACS have little diagnostic value, while several atypical features of the HPI provide significant assistance in identifying patients with cardiac causes of chest pain.

As an extension of that study, Greenslade and colleagues in Australia and New Zealand sought to determine whether the HPI and physical examination features associated with ACS and AMI were consistent across multiple patient populations.^22^ They analyzed an existing dataset based on 1,868 patients who presented with chest pain to one of 12 academic ED across the Asia-Pacific region. Most of the study group—857 patients (45.9%)—were Caucasian, 730 (39.1%) were Chinese, 181 (9.7%) were Korean, and 100 (5.3%) were Indian. ACS was diagnosed in 358 (19.2%) of them. Chinese, Indian, and Korean patients were more likely than Caucasians to report “typical” symptoms (64–66% vs 23%, respectively), but it was only in patients of Indian descent that “typical” symptoms were predictive of ACS (OR=8.82, 95% CI [2.19–35.48]). The presence or absence of symptoms associated with the chest pain was consistently low across the various racial groups. For example, in Chinese patients, the presence of exertional pain (OR=0.41, 95% CI [0.32–0.53]), pleurisy (OR=0.26, 95% CI [0.19–0.35]), nausea (OR=0.52, 95% CI [0.42–0.67]), and shortness of breath (OR=0.59, 95% CI [0.48–0.73]) all decreased the likelihood of ACS. The only physical examination sign that was significantly associated with ACS was diaphoresis, which was true only in Chinese and Caucasian patients. The authors concluded that, although there are some racial differences in symptoms, they do not play a large role given that the HPI and physical examination have little diagnostic value overall for ACS.

## UTILITY OF TRADITIONAL RISK FACTORS FOR CARDIAC DISEASE

The Framingham Heart Study is a landmark longitudinal experiment in population health. Designed by Dr. Thomas Dawber and funded through the National Heart, Lung, and Blood Institute, the purpose of the study was to identify the risk factors associated with cardiovascular disease.^23^ The classic factors are hypertension, hyperlipidemia, smoking, diabetes, age, family history of early cardiac events, and male gender,^24^ with human immunodeficiency virus infection emerging as a new risk factor.^25^ These epidemiologic factors for coronary artery disease (CAD) have traditionally been used in the ED to help determine the likelihood of whether or not a given patient with chest pain had ACS.^13^,^14^,^26^ This application is based on the assumptions that patients with CAD are more likely to have ACS and that population-level factors can be extrapolated to an individual patient. Several studies have examined the value of these risk factors in the ED evaluation of patients with chest pain.

Jayes and associates prospectively collected data from 5,773 patients evaluated in the EDs of six hospitals, who had the typical symptoms suggestive of ischemic disease.^27^ For the 1,743 who did not have clinically obvious coronary disease, medical histories and traditional risk factors were recorded. In male patients, only a history of diabetes (relative risk [RR]=2.4, 95% CI [1.2–4.8]) or family history of myocardial infarction (RR=2.1, 95% CI [1.4–3.3]) significantly increased the risk of ACS. None of the classic risk factors assisted in the risk stratification of female patients. In this population, the magnitude of the RRs associated with these historical risk factors was small compared with those calculated for a simple complaint of chest pain (RR=12.1), an abnormal ST-segment (RR=8.7), or an abnormal T wave on the ECG (RR=5.3). The authors concluded that traditional risk factors had little weight in the overall assessment of ED chest-pain patients for acute cardiac ischemia, especially compared with the chief complaint and the ECG.

This question was revisited by Han and co-workers through a post-hoc analysis of the Internet Tracking Registry of Acute Coronary Syndromes (i*trACS).^28^ Their study included risk factor data from 10,806 patients during their first visit to a U.S. ED for suspected ACS. Cocaine and methamphetamine users as well as those with incomplete records were excluded. ACS was defined as a composite endpoint of death or revascularization within 30 days, diagnostic-related group codes, or positive cardiac markers (creatine kinase [CK-MB] or cardiac troponin) on index hospitalization and was present in 8.1% of the study population. Age was a strong risk factor in this group, so the investigators stratified the results into three groups: <40 years, 40 to 65 years, and >65 years. In those younger than 40, having no risk factors had a negative likelihood ratio (−LR) of 0.17 (95% CI [0.04–0.66]), while having more than four factors had a positive likelihood ratio (LR+) of 7.39 (95% CI [3.09–17.67]). In the intermediate age category, having no risk factors had a −LR of 0.53 (95% CI [0.40–0.71]), and having four or more risk factors had a LR+ of 2.13 (95% CI [1.66–2.73]). In those over the age of 65, having no factors had a −LR of 0.96 (95% CI [0.74–1.23]); the presence of four or more factors had a LR+ of 1.09 (95% CI [0.64–1.62]). The authors concluded that their observations provide evidence for an age-related decrease in the utility of traditional risk factors in judging the likelihood that an ED patient has ACS.

In 2008, Body and associates enrolled a study population of 796 patients over the age of 25 who presented to their university-affiliated ED with suspected cardiac chest pain.^29^ The subjects’ risk factors and hospital course were recorded, and all patients were followed up at six months. Nineteen percent of them met the AMI criteria set forth by the American Heart Association and the European Society of Cardiology, and 12% of those with AMI had no risk factors for cardiac disease. There was no correlation between increasing number of risk factors and increasing incidence of AMI (Table). The area under the receiver operating curve (AUROC) for cardiac risk factors was 0.59 and the risk factor burden was no better than chance for predicting AMI (p=0.59). Univariate logistic regression analysis of the individual risk factors found that smoking history had the strongest association with AMI, but even this had a small OR of 2.31 (95% CI [1.60–3.23]). The authors concluded that traditional risk factors for coronary heart disease were not clinically useful for predicting which patients had AMI in the ED.

Hess and his research team described the presentations of 2,718 patients assessed in three academic EDs between 2007 and 2010, looking at in-hospital and cardiac events 30 days after discharge.^30^ They collected extensive information on subjects, ranging from past medical history, the history of present illness, ECG findings, and patient outcomes (ACS, AMI, revascularization, in-hospital mortality, and death after discharge). Factors in the history that were most predictive of ACS were pain similar to a previous episode of ACS (OR=3.35, 95% CI [2.65–4.24]), radiation to both arms (OR=2.82, 95% CI [1.91–4.17]), worsening chest pain with exertion (OR=2.81, 95% CI [2.23–3.54]), and a change in the pattern of usual angina over the prior 24 hours (OR=2.39, 95% CI [1.83–3.12]). Pain that was pleuritic (OR=0.31, 95% CI [0.20–0.48]), sharp in quality (OR=0.42, 95% CI [0.30–0.59]), or gradual in onset (OR=0.66, 95% CI [0.50–0.87]) decreased the likelihood of ACS. Hypercholesterolemia (OR=2.35, 95% CI [1.83–3.02]), hypertension (OR=2.00, 95% CI [1.56–2.58]), diabetes (OR=1.75, 95% CI [1.35–2.25]), and smoking (OR=1.33, 95% CI [1.06–1.67]) were all weakly predictive of ACS. Known CAD (OR=3.25, 95% CI [2.57–4.11]), angina (OR=2.87, 95% CI [2.26–3.64]), previous AMI (OR=2.14, 95% CI [1.68–2.72]), and peripheral vascular disease (OR=1.99, 95% CI [1.14–3.49]) also increased the likelihood of ACS.

One question arises out of these investigations: why have atypical symptoms become more important than in previous studies? Part of the answer is that the definition of cardiac disease has changed as the technology used to detect it has improved. For example, CK/CK-MB testing is no longer part of the ED workup because it is insensitive compared with troponin testing,^31^ and the presence of a Q wave is not used to determine the management of AMI.^32^ As troponin testing has become increasingly sensitive, clinicians are detecting more mild disease, so some patients who would have been diagnosed with unstable angina in the past are now considered to have NSTEMI.^33^,^34^ Ndrepepa and associates requested simultaneous conventional and ultra-high-sensitivity troponin testing of ED patients with chest pain. They observed that a small amount of cardiac troponin T, detectable only with the high-sensitivity process, was a powerful predictor of long-term all-cause and cardiac mortality and supported reliable stratification of mortality risk in patients with CAD.^34^ However, the presence of small amounts of troponin did not predict nonfatal MI, stroke, or the need for revascularization. The high-sensitivity assay extends the prognostic value of troponin measurements to patients with symptomatic CAD, for whom conventional assays are insensitive. The detection of elevated levels of cardiac troponin T with high-sensitivity assays in samples from patients who do not have myocardial necrosis indicates an adverse cardiovascular risk profile and can be used as an index of cardiovascular risk in general.

Assuming no significant change in the underlying prevalence of cardiac disease, these results suggest that enhanced technologies allow clinicians to detect larger and larger proportions of patients with the disease, beyond the “textbook” cases to the atypical and protean presentations. As the definition of disease widens and a resulting increase in number of patients with cardiovascular disease occurs, the spectrum of clinical presentations must also change. Recent studies of ED patients with chest pain, with a newer emphasis on the predictive power of atypical symptoms, likely reflect this wider range of detectable disease and presentations.

## THE (IN)ACCURACY OF PHYSICAN GESTALT

One response to the poor ability of the HPI and physical exam to identify ACS is to argue that clinician gestalt—a physician’s accumulation of experience combined with data gathered during a patient encounter—is still reliable for this assessment. To assess the accuracy of clinicians’ sense of the diagnosis and outcome, Kline and colleagues retrospectively examined a cohort of adults who came to an ED with complaints of shortness of breath or chest pain.^35^ The investigators excluded patients whose ECGs showed evidence of acute ischemia or infarction; those thought to require admission by the treating physician; and those with serious physical features such as shock, altered mental status, hemorrhage, sepsis, or arrhythmia. The treating clinicians documented their assessment of the cause of patients’ symptoms, and those notes were stratified by level of experience and training (board-certified emergency medicine faculty, third-year resident physicians, and physician assistants). ACS was ultimately diagnosed in 23 (2.7%) of the 840 subjects enrolled. Clinician assessments were stratified as being completed by board-certified physicians (n=560 [67%]), senior residents (202 [24%]), and physician assistants (78 [9%]). Clinician gestalt had a weak correlation with ultimate diagnoses at follow-up (Spearman rho=0.41, 95% CI [0.35–0.47]). The AUROC of gestalt for ACS was somewhat better than chance, at 0.64 (95% CI [0.51–0.77]). The three clinician groups had similar levels of accuracy. The poor overall performance of gestalt in this study was thought to be a result of over-testing and low specificity.

Another investigation by Body and associates prospectively enrolled 458 ED patients presenting with suspected cardiac chest pain, 81 (17.7%) of whom had AMI.^36^ By 30-day follow-up, an additional 19 patients had experienced a major adverse cardiac event (death, AMI, or catheterization). Treating physicians were asked to record their gestalt at the time of presentation on a five-point Likert scale: definitely not ACS, probably not ACS, not sure, probably ACS, and definitely ACS. Clinician gestalt had an AUROC of 0.76 (95% CI [0.70–0.82]). Admitting any patient for whom the probability of ACS was marked as definite, probable, or not sure by the treating physician (i.e., discharging everyone in whom the diagnosis was felt to be probably not or definitely not ACS) had a high sensitivity (95.1%) but low specificity (31.8%) for AMI. Adding a troponin and an ECG to clinician gestalt increased the sensitivity to 100%, though specificity decreased somewhat (28.0%). When a high-sensitivity troponin and an ECG were added when clinicians thought the chest pain was definitely or probably caused by cardiac ischemia, the sensitivity remained high (100%) with an improvement in specificity (46.6%). The authors concluded that gestalt had moderate ability to correctly identify patients with ACS and that, when added to an ECG and cardiac troponin (using a contemporary or high-sensitivity assay), it could identify a proportion of patients at very low risk for ACS (23.1% and 41.7%, respectively).

Carlton and colleagues focused on clinician gestalt where it might help the most—in the assessment of patients with a non-diagnostic ECG.^37^ They enrolled 912 patients with chest pain and a non-diagnostic ECG in an academic ED. Treating physicians were asked to rate the “typicality” of each patient’s chest pain for the diagnosis of ACS and to indicate their level of experience (novice [<1 year of experience] vs experienced [>2 years of practice]). The typicality of the patient’s chest pain had low correlation with a final diagnosis of ACS: the AUROC for both novice and experienced providers was 0.54 to 0.55 (p<0.05 for both). This did not change when the physicians examined patients found to have significant CAD on catheterization. The AUROC for both experienced and inexperienced clinicians was low (AUROC: 0.43–0.56, p>0.05 for all comparisons). The researchers concluded that clinicians’ judgment is of little diagnostic value compared with the ECG and troponins and recommended that future work should focus on high-sensitivity assays and rapid rule-out protocols to accurately identify very low-risk and no-risk patients who may be discharged safely from the ED.

## CASE RESOLUTION AND DISCUSSION

The patient had no prior medical problems and had experienced central, indigestion-like pain that was acute in onset. From the Table, the LR+ are 1.3, 1.0, and 1.1 (respectively). To obtain the total effect of several LRs, we multiply the component LRs: 1.3 x 1.0 x 1.1 = LR_tot_ = 1.43. Using 0.16 as our pre-test probability of disease (AMI, cardiac revascularization, or death),^30^ our post-test probability of disease becomes 0.27. Even with a relatively benign story and no cardiac risk factors, this patient will require further testing before he can be discharged safely. Adding in our patient’s normal ECG (LR+ = 0.2) does lower his risk, and our new post-test probability of disease is still 0.06. The patient in the vignette did well, but most physicians would feel that a 6% probability of ACS is too high.^38^

To be considered a “strong” test with the ability to rule in or rule out diagnoses, a test should have LRs greater than 10, or smaller than 0.1. LRs of this size will generally alter the post-test probability of disease by 45%.^38^ To be of any clinical utility, tests should at least have LRs greater than 3, or less than 0.4. Note that tests that perform at this level will only change the post-test probability of disease by about 20%.^39^ As can be seen from the Table, very few factors meet even this lower threshold. Of those features that have greater predictive value, some are rare (e.g., hypotension) and therefore don’t apply to most ED patients. In practice, we know that it is the accumulation of many small factors that tips our internal balance, indicating when it is worthwhile to pursue a particular diagnosis. But in the case of chest pain and ACS, a patient may have many negative predictors of ACS, or have no positive predictors for ACS, yet their remaining risk may still be higher than what many clinicians would accept (i.e., 1% or less).^38^ Unless there is a clear alternative cause, further testing is virtually required in all ED patients with chest pain. Therefore, despite their prominence in international guidelines, the HPI, Framingham risk factors, and physician gestalt all appear to have limited value for “ruling in” or “ruling out” ACS.

## CONCLUSION

A few factors are consistently associated with an increased likelihood of ACS: pain accompanied by diaphoresis or vomiting, radiation of the pain (especially to both arms), and pain aggravated by exertion. Similarly, the features that decreased the risk of ACS were reproducible chest wall tenderness, or pain that was pleuritic, sharp, or positional. These features are useful in identifying a low- or no-risk subgroup of ED patients with chest pain as a part of a rational rule-out strategy that includes troponin measurement and ECG testing. Acute care providers should strongly consider these factors when risk stratifying patients with chest pain.

## Figures and Tables

**Table:** Characteristics of each predictive clinical feature as a diagnostic test for ACS in the emergency department.

Predictor	Sensitivity (%)	Specificity (%)	PPV^1^ (%)	NPV^2^ (%)	LR+^3^	LR−4	Reference
Pain Characteristics							
Chest pain	56.8	33.5	10.8	84.6	0.85	1.3	a
	70.2	42.1	45.2	67.5	1.3	0.9	b
Pain radiates to both shoulders/arms	13.5	94.8	37.0	82.8	2.3	0.9	c
Pain radiates to right shoulder/arm	18.9	91.8	34.6	83.2	2.6	0.9	c
Neck/jaw pain	23.5	84.8	18.0	88.7	1.6	0.9	a
	14.9	90.2	50.8	60.9	1.5	0.9	b
Back pain	11.6	86.7	11.0	87.4	0.9	1.0	a
	6.5	93.0	38.9	59.4	0.9	1.0	b
Central pain	85.1	34.1	22.8	91.0	1.3	0.4	c
Sharp quality	11.9	75.4	6.4	85.9	0.5	1.2	a
Pleuritic	6.5	81.5	4.8	86.1	0.4	1.1	a
Timing of the pain							
Acute onset (<1 hr)	75.9	32.3	13.7	90.5	1.1	0.7	a
Gradual onset (>1 hr)	21.1	71.2	9.4	86.5	0.7	1.1	a
Worse with exertion	53.3	71.1	20.6	91.5	1.8	0.7	a
Change in pattern of stable angina	27.4	86.4	22.1	89.4	2.0	0.8	a
Associated symptoms							
Diaphoresis	28.3	79.2	16.1	88.7	1.4	0.9	a
	25.1	81.6	48.2	61.6	1.4	0.9	b
	36.5	94.3	22.9	85.4	6.4	0.7	c
Reported vomiting	21.1	76.9	11.4	87.4	0.9	1.0	a
	21.9	79.7	42.3	60.0	1.1	1.0	b
	16.2	94.8	41.4	83.2	3.1	0.9	c
Dyspnea	47.0	61.3	14.6	89.1	1.2	0.9	a
	41.9	62.0	42.9	61.1	1.1	0.9	b
Palpitations	6.0	91.5	32.5	58.9	0.7	1.0	b
Fatigue	13.0	85.8	38.4	59.2	0.9	1.0	b
Indigestion	15.8	84.5	41.0	59.6	1.0	1.0	b
Dizziness/faintness	19.5	73.4	33.3	57.3	0.7	1.1	b
Hypotension	6.8	97.7	40.0	82.1	3.0	1.0	c
ECG Findings							
Acute ischemic ECG changes	71.0	81.3	46.5	92.5	3.8	0.4	c
ST-segment depression >0.5 mm	17.3	97.2	46.4	89.3	6.1	0.9	a
T-wave inversion	14.9	93.9	25.6	88.7	2.4	0.9	a
Left bundle-branch block	7.1	97.2	26.4	88.1	2.5	1.0	a
Right bundle-branch block	5.4	95.8	15.3	87.8	1.3	1.0	a
Q waves	11.6	91.3	15.8	88.0	1.3	1.0	a
Number of Risk Factors							
≥1	92.6	12.2	23.0	83.1	1.1	0.6	d
	95.2	9.8	6.8	91.4	1.1	0.5	e
≥2	58.1	37.0	19.0	81.6	0.9	1.1	d
	80.7	29.6	9.0	92.3	1.1	0.7	e
≥3	27.7	66.7	13.6	80.0	0.8	1.1	d
	53.0	60.9	10.7	92.4	1.4	0.8	e
≥4	11.5	90.3	21.3	81.7	1.2	1.0	d
	20.4	88.1	15.1	92.3	1.7	0.9	e

1 PPV refers to positive predictive value, the probability of disease given a positive test and the study’s disease prevalence.

2 NPV refers to negative predictive value, the probability of not having disease given a negative test result and the study’s disease prevalence.

3 Positive likelihood ratio, the change in probability of disease when the related feature is present.

4 Refers to negative likelihood ratio, the change in probability of disease when the stated feature is absent.

a Hess EP, Brison RJ, Perry JJ, et al. Development of a clinical prediction rule for 30-day cardiac events in emergency department patients with chest pain and possible acute coronary syndrome. *Ann Emerg Med*. 2012;59(2):115–25.

b Milner KA, Funk M, Richards S, Vaccarino V, Krumholz HM. Symptom predictors of acute coronary syndromes in younger and older patients. *Nursing Res*. 2001;50(4):233–41.

c Body R, Carley S, Wibberley C, et al. The value of symptoms and signs in the emergent diagnosis of acute coronary syndromes. *Resuscitation*. 2010;81(3):281.

d Body R, McDowell G, Carley S, Mackway-Jones K. Do risk factors for chronic coronary heart disease help diagnose acute myocardial infarction in the Emergency Department? *Resuscitation*. 2008;79(1):41–5.

e Han JH, Lindsell CJ, Storrow AB, et al. The role of cardiac risk factor burden in diagnosing acute coronary syndromes in the emergency department setting. *Ann Emerg Med*. 2007;49(2):145–52.
